# An EPIC predictor of gestational age and its application to newborns conceived by assisted reproductive technologies

**DOI:** 10.1186/s13148-021-01055-z

**Published:** 2021-04-19

**Authors:** Kristine L. Haftorn, Yunsung Lee, William R. P. Denault, Christian M. Page, Haakon E. Nustad, Robert Lyle, Håkon K. Gjessing, Anni Malmberg, Maria C. Magnus, Øyvind Næss, Darina Czamara, Katri Räikkönen, Jari Lahti, Per Magnus, Siri E. Håberg, Astanand Jugessur, Jon Bohlin

**Affiliations:** 1 Department of Genetics and Bioinformatics, Norwegian Institute of Public Health, Oslo, Norway; 2 Centre for Fertility and Health, Norwegian Institute of Public Health, Oslo, Norway; 3 Institute of Health and Society, University of Oslo, Oslo, Norway; 4 Department of Global Public Health and Primary Care, University of Bergen, Bergen, Norway; 5 Department of Mathematics, University of Oslo, Oslo, Norway; 6 Deepinsight, Karl Johans Gate 8, Oslo, Norway; 7 Department of Medical Genetics, Oslo University Hospital, Oslo, Norway; 8 Department of Psychology and Logopedics, Faculty of Medicine, University of Helsinki, Helsinki, Finland; 9 MRC Integrative Epidemiology Unit, University of Bristol, Bristol, UK; 10 Bristol Medical School, Population Health Sciences, Bristol, UK; 11 Division of Mental and Physical Health, Norwegian Institute of Public Health, Oslo, Norway; 12 Department of Translational Research in Psychiatry, Max-Planck-Institute of Psychiatry, Munich, Germany; 13 Division for Infection Control and Environmental Health, Department of Infectious Disease Epidemiology and Modelling, Norwegian Institute of Public Health, Oslo, Norway

**Keywords:** DNA methylation, Epigenetics, Gestational age, Illumina MethylationEPIC BeadChip, Assisted reproductive technologies, IVF, ICSI, MoBa, MBRN, PREDO

## Abstract

**Background:**

Gestational age is a useful proxy for assessing developmental maturity, but correct estimation of gestational age is difficult using clinical measures. DNA methylation at birth has proven to be an accurate predictor of gestational age. Previous predictors of epigenetic gestational age were based on DNA methylation data from the Illumina HumanMethylation 27 K or 450 K array, which have subsequently been replaced by the Illumina MethylationEPIC 850 K array (EPIC). Our aims here were to build an epigenetic gestational age clock specific for the EPIC array and to evaluate its precision and accuracy using the embryo transfer date of newborns from the largest EPIC-derived dataset to date on assisted reproductive technologies (ART).

**Methods:**

We built an epigenetic gestational age clock using Lasso regression trained on 755 randomly selected non-ART newborns from the Norwegian Study of Assisted Reproductive Technologies (START)—a substudy of the Norwegian Mother, Father, and Child Cohort Study (MoBa). For the ART-conceived newborns, the START dataset had detailed information on the embryo transfer date and the specific ART procedure used for conception. The predicted gestational age was compared to clinically estimated gestational age in 200 non-ART and 838 ART newborns using MM-type robust regression. The performance of the clock was compared to previously published gestational age clocks in an independent replication sample of 148 newborns from the Prediction and Prevention of Preeclampsia and Intrauterine Growth Restrictions (PREDO) study—a prospective pregnancy cohort of Finnish women.

**Results:**

Our new epigenetic gestational age clock showed higher precision and accuracy in predicting gestational age than previous gestational age clocks (*R*^2^ = 0.724, median absolute deviation (MAD) = 3.14 days). Restricting the analysis to CpGs shared between 450 K and EPIC did not reduce the precision of the clock. Furthermore, validating the clock on ART newborns with known embryo transfer date confirmed that DNA methylation is an accurate predictor of gestational age (*R*^2^ = 0.767, MAD = 3.7 days).

**Conclusions:**

We present the first EPIC-based predictor of gestational age and demonstrate its robustness and precision in ART and non-ART newborns. As more datasets are being generated on the EPIC platform, this clock will be valuable in studies using gestational age to assess neonatal development.

**Supplementary Information:**

The online version contains supplementary material available at 10.1186/s13148-021-01055-z.

## Background

Accurate determination of gestational age is important for assessing fetal development and maturity. This is necessary for investigating the impact of prenatal factors on pregnancy outcomes and any deviation from normal fetal development [1, 2]. Although gestational age at birth exhibits some normal variation, both preterm and post-term births are associated with an increased risk of adverse perinatal outcomes and health outcomes later in life [3–7]. The effects of gestational age at birth on health outcomes may be linked to epigenetic patterns established in utero or early in the postnatal period [8, 9]. Changes in these patterns may interfere with critical developmental processes [10–12] and trigger phenotypic changes that persist throughout life. This may be even more pertinent to children conceived by assisted reproductive technologies (ART), because ART procedures coincide with the extensive epigenetic reprogramming in the early embryo [13, 14].

DNA methylation (DNAm) is the most studied epigenetic mark in humans. It has, in recent years, been used to build gestational age clocks that can predict gestational age [15–18]. Earlier clocks were built using DNAm data from the Illumina HumanMethylation27 (27 K) or the Illumina HumanMethylation450 (450 K) BeadChip arrays, both of which have subsequently been replaced by the Illumina MethylationEPIC BeadChip (EPIC). EPIC has nearly twice (865,859 CpGs) as many CpGs as 450 K, and a stronger focus on regulatory elements [19]. Although EPIC includes over 90% of the probes on 450 K [19], six to eight of the CpGs included in existing gestational age clocks are not present on EPIC. This discrepancy may affect the precision of the published clocks in predicting gestational age when applied to DNAm data generated on EPIC [20]. Therefore, it is essential to develop a new gestational age clock that is updated and optimized for EPIC. Equally important is to elucidate whether the additional CpGs on EPIC enhance gestational age prediction.

A challenge in developing accurate gestational age clocks is the lack of information on the exact gestational age of the newborns. The standard approaches for estimating gestational age, based on ultrasound measurements or the last menstrual period (LMP), have thus far been used for training and testing epigenetic clocks. Ultrasound and LMP are widely used in clinical settings and have their individual advantages and limitations. While LMP can be informative, it suffers from large variability, in part due to varying length of the follicular phase. Ultrasound is much more precise but still depends on the size of the fetus at the time of ultrasound [1, 21, 22]. On the other hand, for children conceived by ART, the exact time when the embryo is transferred back to the uterus is known. Although there may be some differences in the days before fertilization and embryo transfer, and the developmental speed may differ in the in vitro setting, the embryo transfer date (ETD) provides a more direct estimate of gestational age [23]. Therefore, DNAm data from ART births is particularly advantageous for developing and validating gestational age clocks. To our knowledge, no gestational age clock has yet been developed using ETD, although its use has been called for previously [16].

In addition to gestational age prediction, gestational age clocks can be used to estimate gestational age acceleration (GAA), which is defined as the discrepancy between gestational age predicted from DNAm data and gestational age derived from clinical measurements [16, 24]. Investigating GAA is important because of its reported association with several measures related to birth outcomes, such as the cerebroplacental ratio (a robust indicator of prenatal stress [25]), higher maternal body mass index, and larger birth size [26]. Although children conceived by ART have a higher risk of spontaneous preterm birth [27] and other adverse perinatal outcomes [28–30], only one small study has explored GAA in ART children [31].

To address these knowledge gaps, we developed a new gestational age clock based on EPIC-derived DNAm data from newborns in the Norwegian Study of Assisted Reproductive Technologies (START), which is a substudy within the Norwegian Mother, Father and Child Cohort Study (MoBa) [32]. We validated this clock in test sets of ART and non-ART newborns in START, and also in an external dataset from the Finnish Prediction and Prevention of Preeclampsia and Intrauterine Growth Restriction (PREDO) study [33], which was used as a replication cohort. We also used the new EPIC-based clock to explore differences in GAA between ART and non-ART newborns.

## Results

### The EPIC gestational age clock

**Fig. 1:**
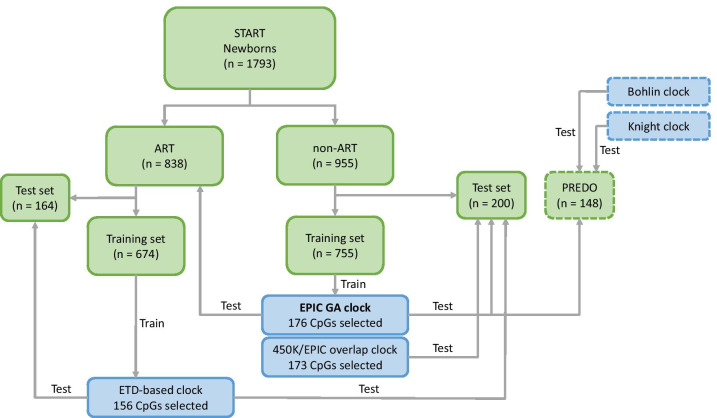
Analysis flow. START newborns were grouped into ART and non-ART, and each group was randomly assigned to a training and test set. The non-ART training set was used to develop the EPIC GA clock and the 450 K/EPIC overlap clock. The ART training set was used to develop the ETD-based clock. All three clocks were tested in the non-ART test set. The EPIC GA clock, the Bohlin clock, and the Knight clock were also tested in the PREDO test set. The datasets are marked in green, and the clocks are marked in blue. START-derived datasets and clocks are marked with solid lines. External datasets and clocks are marked with dashed lines

Table 1 and Fig. 1 provide overviews of the datasets used in this study. We fit a least absolute shrinkage and selection operator (Lasso) regression on DNAm data from 755 non-ART newborns in START. 176 CpGs were selected for being predictive of gestational age. Individual CpG sites and their corresponding coefficients are provided in Additional file 4.

**Table 1:** Characteristics of the datasets used to evaluate the EPIC GA clock

Dataset	N	GA range (US, days)	Median GA (US, days)	GA range (ETD, days)	Median GA (ETD, days)	Sex ratio (% male)
START non-ART						
Training set	755	216–299	281.1	–	–	49
Test set	200	228–300	281.3	–	–	46
START ART						
Total	838	218–301	280.4	214–302	280.4	53
Training set	674	228–300	280.3	227–302	280.3	53
Test set	164	218–301	280.8	214–298	280.8	54
PREDO non-ART						
Test set	148	227–296	278.9	–	–	51

*GA* gestational age, *US* ultrasound, *ETD* embryo transfer date

**Fig. 2:**
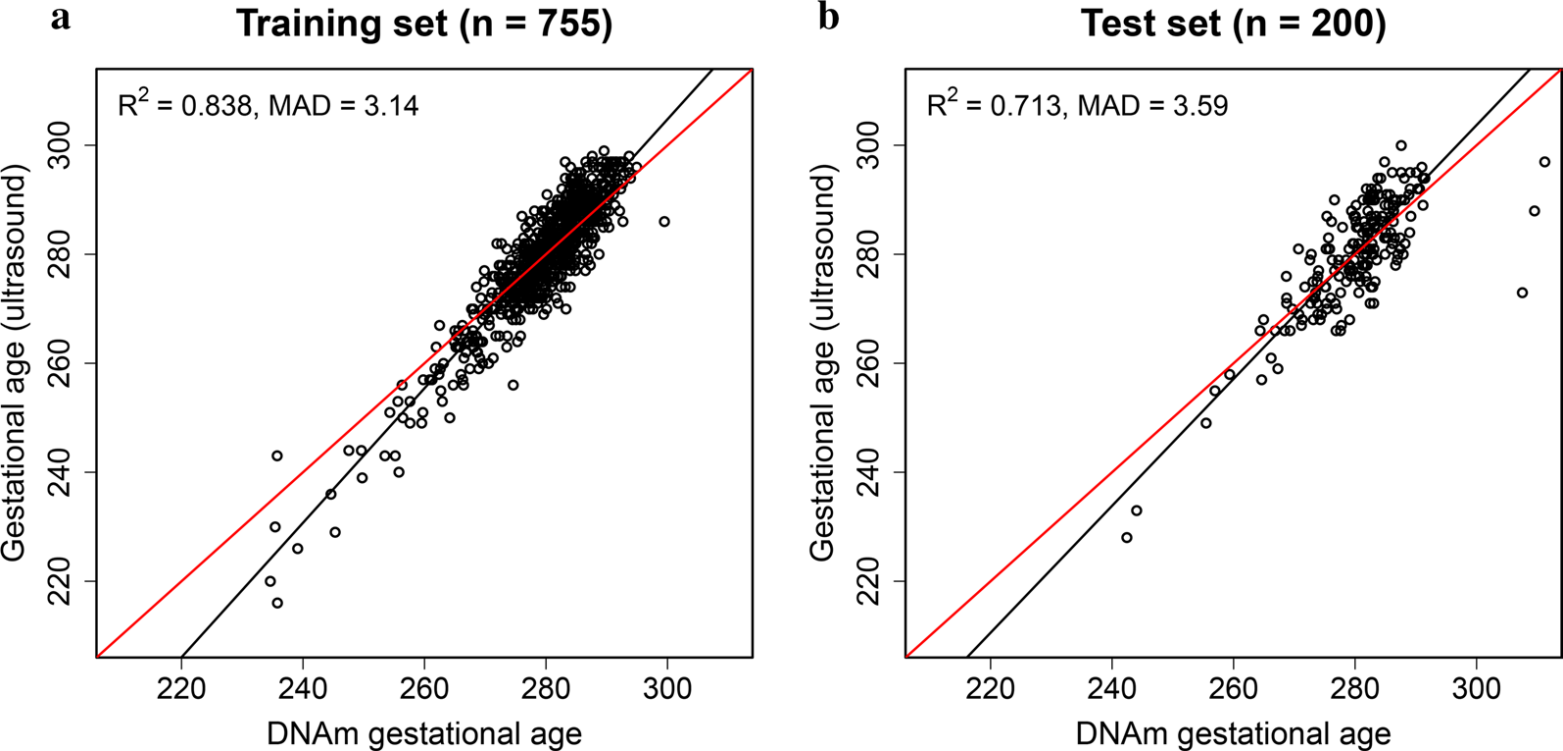
Using the EPIC GA clock to predict gestational age. Panel **a** shows the scatter plot of predicted gestational age against gestational age estimated by ultrasound in the training set (*n* = 755). Panel **b** shows the corresponding predicted gestational age in the test set (*n* = 200). The red line indicates a perfect correlation between DNAm-based gestational age and ultrasound-based gestational age. The black line indicates the MM-type robust regression of ultrasound-based gestational age on DNAm-based gestational age

We validated the resulting predictor, referred to as “EPIC GA clock” hereafter, in a test set of 200 non-ART newborns from START. The EPIC GA clock showed an *R*^2^ of 0.713 and a median absolute deviation (MAD) of 3.59 days (Fig. 2, Table 2).

**Table 2:** Results of gestational age prediction in START and PREDO

Dataset* (count)	GA estimation method	Clock	R^2^	SE	MAD
START non-ART (*n* = 200)	Ultrasound	EPIC GA clock	0.713	5.52	3.59
	Ultrasound	450 K/EPIC overlap clock	0.691	5.81	3.75
	Ultrasound	ETD-based clock	0.668	6.08	4.24
PREDO non-ART (*n* = 148)	Ultrasound	EPIC GA clock	0.724	5.08	3.42
	Ultrasound	Bohlin clock	0.610	6.06	6.69
	Ultrasound	Knight clock	0.406	6.99	4.55
START ART (*n* = 838)	Ultrasound	EPIC GA clock	0.767	5.32	3.80
	ETD	EPIC GA clock	0.767	5.30	3.70

*See also Table 1 and Fig. 1 for further details on these datasets

*GA* gestational age, *SE* standard error, *MAD* median absolute deviation, *ETD* embryo transfer date

### Comparison with previously published gestational age clocks in an external replication cohort (PREDO)

**Fig. 3:**
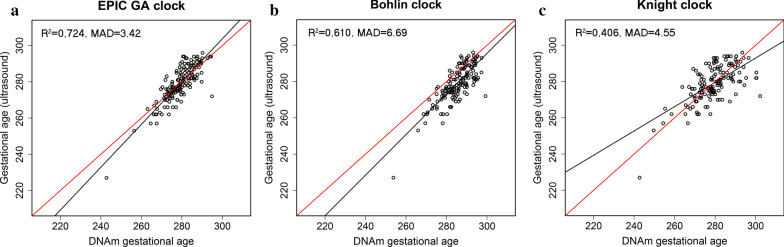
Prediction of gestational age in the PREDO non-ART dataset (*n* = 148). Panel **a** shows the scatter plot of predicted gestational age against gestational age estimated by ultrasound using the EPIC GA clock. The corresponding predictions using the Bohlin clock and the Knight clock are shown in panel **b** and **c**, respectively. The red line indicates a perfect correlation between DNAm-based gestational age and ultrasound-based gestational age. The black line indicates the MM-type robust regression of ultrasound-based gestational age on DNAm-based gestational age

Using an external dataset of EPIC-derived DNAm data on 148 non-ART newborns from the PREDO study [33], we compared the performance of our EPIC GA clock with two published epigenetic gestational age clocks that were built on DNAm data from the previous methylation arrays: the Bohlin clock [15], based on 450 K, and the Knight clock [16], based on 27 K and 450 K. Eight CpGs in the Bohlin clock and six CpGs in the Knight clock were absent from the PREDO dataset and were thus excluded from the analysis. Compared to the Bohlin and Knight clocks, our EPIC GA clock showed higher precision and accuracy in predicting gestational age (Fig. 3, Table 3). The difference in *R*^2^ between the Bohlin clock and the EPIC GA clock was -0.062 (95% confidence interval (CI): −0.117, −0.014), and the difference in MAD was 3.27 days (95% CI: 1.87, 3.92). The corresponding statistics for the Knight clock versus our EPIC GA clock were -0.247 (95% CI: −0.342, −0.161) for *R*^2^ and 1.13 days (95% CI: 0.196, 2.40) for MAD.

**Table 3:** Bootstrapped differences in *R*^2^, SE, and MAD between different clocks and GA estimation methods

Dataset * (count)	Comparison between clocks	R^2^ (95% CI)	SE (95% CI)	MAD (95% CI)
START non-ART (*n* = 200)	450 K/EPIC overlap – EPIC GA	−0.0001 (−0.021, 0.018)	0.001 (−0.142, 0.175)	0.162 (−0.375, 0.794)
	ETD-based – EPIC GA	0.048 (−0.041, 0.123)	−0.409 (−1.00, 0.335)	0.645 (−0.181, 1.209)
	ETD-based – 450 K/EPIC overlap	0.048 (−0.039, 0.119)	−0.410 (−1.03, 0.308)	0.483 (−0.409, 0.984)
PREDO Non-ART (*n* = 148)	Bohlin – EPIC GA	−0.062 (−0.117, −0.014)	0.528 (0.095, 0.994)	3.27 (1.87, 3.92)
	Knight – EPIC GA	−0.247 (−0.342, −0.161)	1.89 (1.97, 2.69)	1.13 (0.196, 2.40)
	Knight – Bohlin	−0.185 (−0.273, −0.102)	1.36 (0.698, 1.97)	−2.15 (−3.11, −0.382)

Dataset * (count)	Comparison between GA estimation methods	*R*^2^ (95% CI)	SE (95% CI)	MAD (95% CI)
START ART (n = 838)	ETD – ultrasound	0.015 (−0.003, 0.033)	−0.284 (−0.544, −0.037)	−0.102 (−0.465, 0.174)

*See Table 1 and Fig. 1 for further details on these datasets

*GA* gestational age, *SE* standard error, *MAD* median absolute deviation, *ETD* embryo transfer date

### Assessing the impact of CpGs unique to EPIC on the prediction of gestational age

**Fig. 4:**
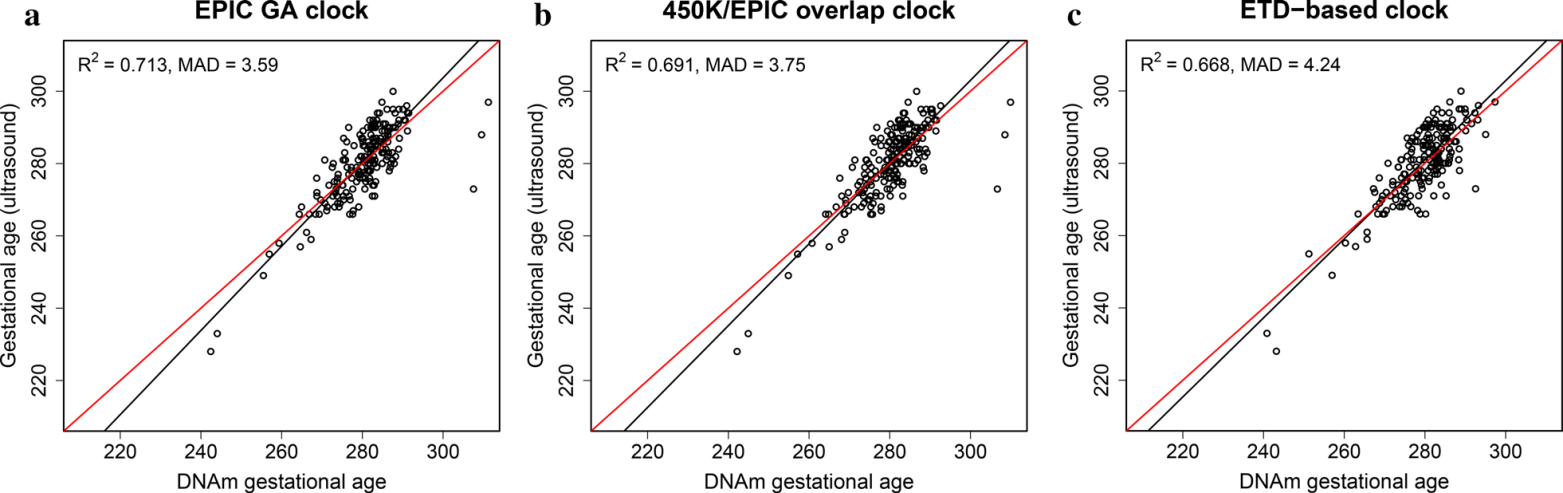
Prediction of gestational age using the EPIC GA, 450 K/EPIC, and ETD-based clocks. Scatter plots of predicted gestational age using (**a**) the EPIC GA clock, (**b**) the 450 K/EPIC overlap clock, and (**c**) the ETD-based clock against gestational age estimated by ultrasound in a test set (n = 200) of non-ART newborns from START. The red line indicates a perfect correlation between DNAm-based gestational age and ultrasound-based gestational age. The black line indicates the MM-type robust regression of ultrasound-based gestational age on DNAm-based gestational age

Of the 176 CpGs selected in the EPIC GA clock, 89 were found exclusively on EPIC. To assess whether the additional CpGs unique to EPIC affect the prediction parameters *R*^2^ and MAD, we built a separate clock using the same training set but this time only including the 397,473 probes that are present on both 450 K and EPIC. We compared the performance of this new “450 K/EPIC overlap clock” (173 CpGs) to the EPIC GA clock (Fig. 4; Table 2) and found no significant difference in *R*^2^ (−0.0001; 95% CI: −0.021, 0.018) or MAD (0.162; 95% CI: −0.375, 0.794) (Table 3). In terms of CpG overlap, 81 CpGs in the 450 K/EPIC overlap clock were also present in the EPIC GA clock.

### Using the embryo transfer date (ETD) to predict gestational age

A great advantage of the ART dataset is that the ETD is known for the ART-conceived children. We thus developed a gestational age clock using the ETD of ART-conceived children to investigate whether it was possible to achieve a better predictor of gestational age. Six hundred and seventy-four ART newborns from START (Table 1, Fig. 1) were used to train the ETD-based clock. Additional file 1: Figure S1 shows the performance of the ETD-based clock for ultrasound- and ETD-estimated gestational age in the START ART training and test set, respectively. When compared to the EPIC GA clock in the non-ART test set from START, the ETD-based clock showed a similar performance, with an R^2^ difference of 0.048 (95% CI: −0.041, 0.123) and a difference in MAD of 0.645 (95% CI: −0.181, 1.209) (Fig. 4; Table 3). The ETD-based GA clock contained 155 CpGs, and only 19 of them were in common with those of the EPIC GA clock.

### Application of the EPIC GA clock to ART children

**Fig. 5:**
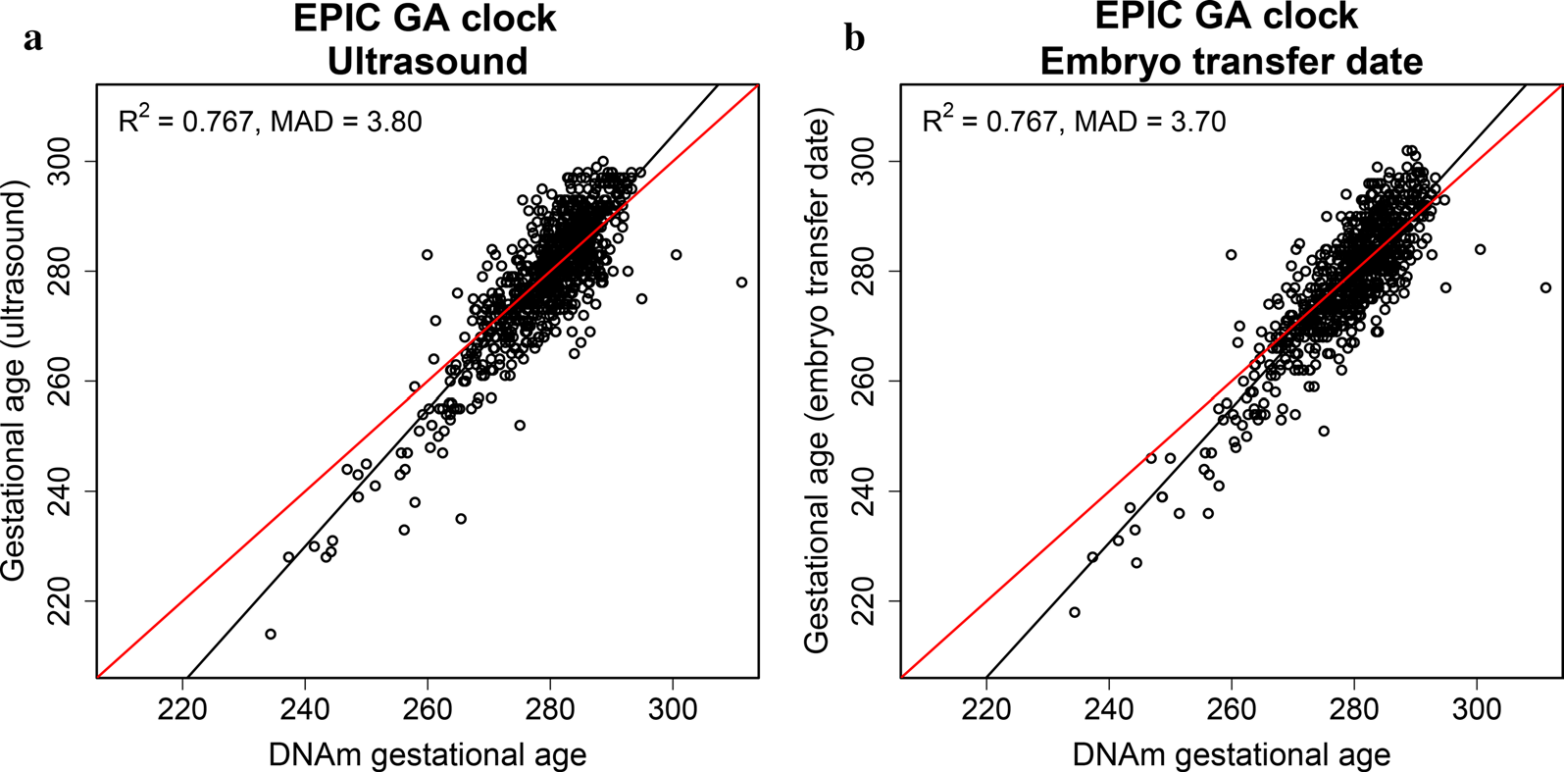
Prediction of gestational age estimated by ultrasound and embryo transfer date (ETD). Scatter plots of predicted gestational age using the EPIC GA clock against gestational age estimated by **a** ultrasound and **b** ETD in a dataset of ART-born children (*n* = 838) in START. The red line indicates a perfect correlation between DNAm-based gestational age and **a** ultrasound-based or **b** ETD-based gestational age. The black line shows the regression of **a** ultrasound-based or **b** ETD-based gestational age on DNAm-based gestational age

To assess the performance of the EPIC GA clock in ART-children, we applied the EPIC GA clock to the cord-blood DNAm data of 838 newborns conceived by ART (Table 1, Fig. 1). We compared predicted gestational age to gestational age estimated by ultrasound measurements and by ETD, respectively (Fig. 5). Gestational age estimated by ultrasound measurement and ETD was predicted with similar precision (*R*^2^ difference of 0.015 (95% CI: −0.003, 0.033); Fig. 5, Table 3) and accuracy (MAD difference of −0.102 (95% CI: −0.465, 0.174)).

### Gestational age acceleration in ART children

**Fig. 6:**
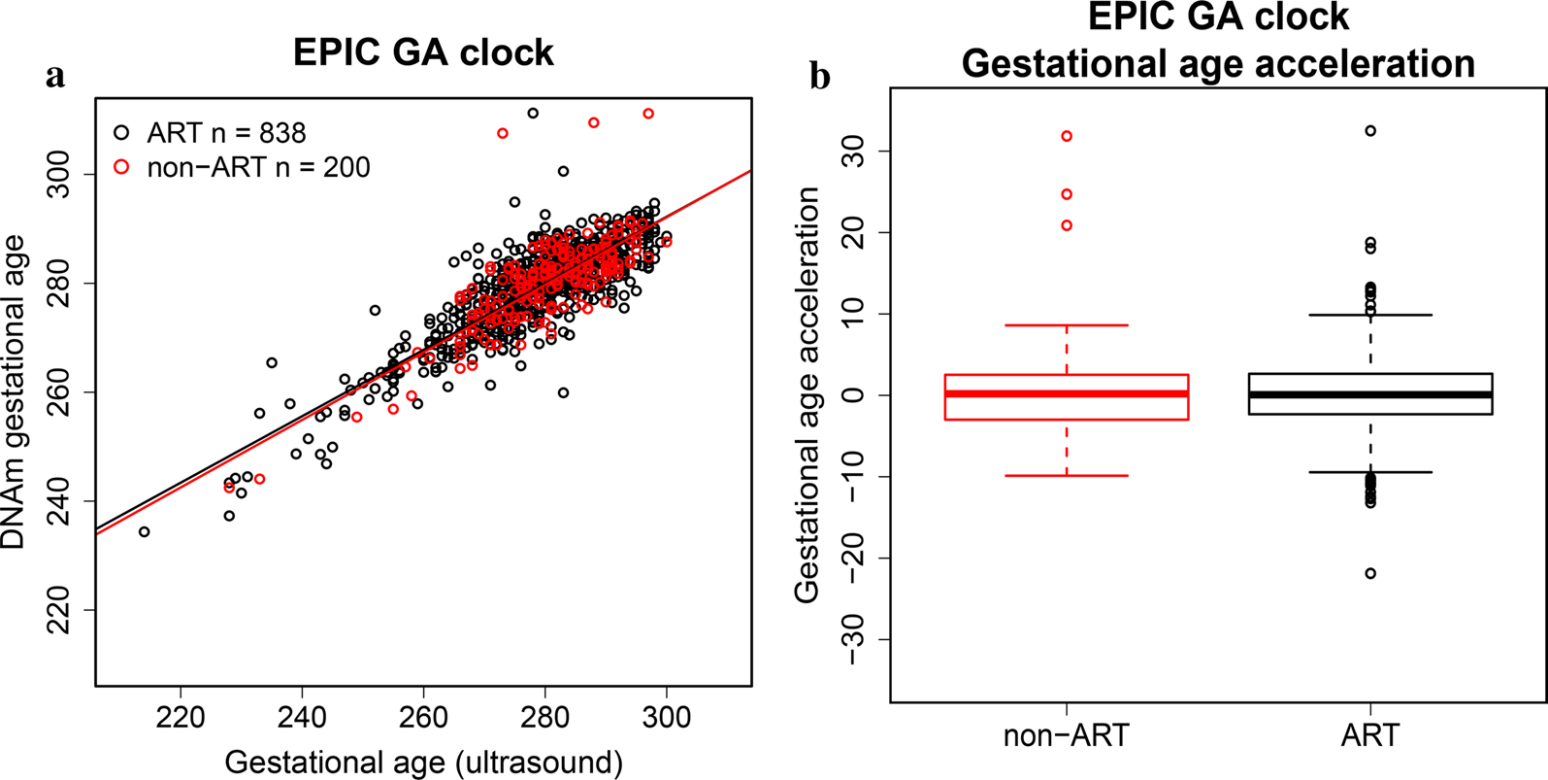
Gestational age and gestational age acceleration (GAA) in ART and non-ART children. Panel **a** shows predicted gestational age using the EPIC GA clock against gestational age estimated by ultrasound in ART (*n* = 838, highlighted in black) and non-ART (*n* = 200, highlighted in red) newborns from START. Panel** b** shows GAA represented by the regressions of EPIC GA clock-predicted gestational age on ultrasound-based gestational age in the ART and non-ART newborns

To assess whether GAA is associated with ART, we first regressed gestational age predicted by the EPIC GA clock on gestational age estimated by ultrasound in 200 non-ART and 838 ART newborns from START. GAA was calculated using the residuals from this regression. Next, we analyzed the relationship between GAA and ART by performing a logistic regression of ART on GAA. We found no significant difference in GAA between the ART (*n* = 838) and non-ART (*n* = 200) newborns (*p* = 0.388, Fig. 6).

Aside from ETD, another major advantage of the START dataset is that the specific ART procedure used for conception was known, i.e., whether in vitro fertilization (IVF) was used alone or together with intracytoplasmic injection of sperm (ICSI), and whether the embryo was transferred fresh or after being frozen. We found no significant difference in GAA between newborns conceived by IVF alone (*n* = 470) and those conceived by IVF in combination with ICSI (*n* = 338) (*p* = 0.976, Additional file 2: Figure S2). Furthermore, there was no significant difference between fresh (*n* = 693) and frozen (*n* = 115) embryo transfer (*p* = 0.274, Additional file 3: Figure S3).

### Gene-enrichment analysis

To explore the biological significance of the 176 CpGs selected in our EPIC GA clock, we performed gene-enrichment analyses of the genes annotated for the selected CpGs. Using the annotation data provided in Illumina’s Infinium MethylationEPIC v1.0 B4 Manifest file, we identified 154 unique gene names annotated for the 176 selected CpGs. A list of the 176 CpGs and their annotated genes is provided in Additional file 4. The software WebGestalt [34] was used to perform gene-enrichment analyses of the 154 genes [35]. WebGestalt identified 78 categories as being significantly enriched at a false discovery rate (FDR) < 0.01. The category with the highest enrichment ratio was “regulation of platelet-derived growth factor receptor signaling pathway,” containing *LRP1*, *HIP1R*, *HGS*, and *SRC* (enrichment ratio = 37; FDR = 0.003). Several of the significant hits were related to abnormal morphology of the eye, ear, nose, and other developmental categories, e.g., “plasma membrane-bounded cell projection organization” and “negative regulation of cellular biosynthetic process.” The complete output of the WebGestalt analyses is provided in Additional file 5.

## Discussion

We present the first EPIC-based predictor of gestational age and demonstrate its robustness and precision in ART versus non-ART newborns. This study benefited greatly from having the largest ART dataset to date, with detailed information on ETD and the specific procedure used for conception. Our EPIC GA clock, trained on the START dataset, outperformed previous cord blood-based gestational age clocks when compared in an independent Finnish test set (PREDO).

Previous DNAm-based clocks were developed using the now outdated 27 K and 450 K. EPIC has almost twice as many CpGs as 450 K, and while 27 K and 450 K mostly cover areas around genes and CpG-islands, some of the additional probes on EPIC target distal regulatory elements and intergenic regions [36]. We, therefore, hypothesized that the additional CpGs unique to EPIC might have enhanced the performance of the EPIC GA clock. However, when we developed a separate clock featuring only those probes that are shared between 450 K and EPIC, we observed a similar performance to the EPIC GA clock, indicating that the additional CpGs on EPIC did not significantly enhance the prediction of gestational age. This observation is consistent with recent findings on age prediction by Lee et al*.* [37]. Another plausible explanation for the superior performance of our EPIC GA clock might be related to the fact that eight CpGs in the Bohlin clock and six CpGs in the Knight clock are absent from the EPIC array. This discrepancy might have reduced the prediction accuracy of the earlier clocks when applied to EPIC data.

A substantial advantage of the START dataset is its large sample size combined with detailed information on ETD for the ART-conceived newborns and the specific ART procedures used for conception. Using ETD provides a more direct estimate of gestational age than estimates based on ultrasound measurement or LMP [23]. We thus checked whether a clock trained on gestational age estimated by ETD would lead to a further improvement in gestational age prediction. The results showed that the two clocks had similar performance, despite the low overlap in CpGs and genes. This suggests that using ETD-based gestational age estimates for training does not significantly enhance prediction compared to clocks trained on ultrasound-based estimates, further highlighting the precision of the EPIC GA clock.

A higher risk of spontaneous preterm birth and other adverse perinatal outcomes has been reported among ART-conceived children [28–30]. Given that the timing of ART procedures coincides with the extensive epigenetic remodeling in the gametes and early embryo, and, further that epigenetic alterations have been reported in ART embryos and children [38–40], we investigated whether the epigenetic gestational age of ART newborns differed significantly from that of non-ART newborns. When we applied the EPIC GA clock to ART newborns, the precision of the gestational age prediction remained similar to that of the non-ART newborns, indicating that the clock is also well suited for predicting gestational age in ART newborns. Furthermore, the EPIC GA clock predicted both ETD-based and ultrasound-based gestational age equally well, again underscoring the precision of the clock. Finally, we found no significant differences in GAA between ART and non-ART newborns.

ART is a collective term used to describe different procedures and categories that may have different impacts on fetal DNAm. It is therefore particularly important to investigate whether gestational age prediction differs according to the specific ART procedure used. For instance, embryos may be transferred to the uterus when they are fresh or after being frozen, and IVF may or may not involve ICSI. A previous study [31] examining GAA in ICSI newborns compared to non-ART newborns did not find any significant difference between the two groups. However, the authors detected a significant decrease in DNAm-predicted gestational age at birth among the ICSI newborns. To verify these findings in our dataset, we conducted another set of analyses to explore differences between IVF, ICSI, and non-ART newborns, as well as between fresh, frozen, and non-ART-conceived newborns. We found no significant differences in DNAm-predicted GA or GAA between any of the groups (Additional file 2: Figure S2 and Additional file 3: Figure S3), further strengthening the hypothesis that GAA is not associated with ART.

Although DNAm is strongly associated with gestational age, the mechanisms underlying this association are not well understood. A closer inspection of the specific CpGs selected for gestational age prediction and the overlap between different clocks may provide some answers. Of the 176 CpGs selected by the EPIC GA clock, only 11 were in common with the CpGs in the Bohlin clock, and none overlapped with the CpGs in the Knight clock. This could partly be explained by the 89 EPIC-specific CpGs. The lack of overlap in CpGs across different clocks has also been observed in age prediction models [41]. Our analyses showed little overlap between the EPIC GA clock and the ETD-based clock, even though both were trained on EPIC data. As Lasso regression and elastic net regression may select CpGs that are not associated with the outcome per se [42], dataset-specific CpGs could end up being included in the model. Furthermore, Lasso selects one CpG for each group of correlated (or neighboring) CpGs, whereas elastic net regression selects several CpGs, leading to a so-called “grouping effect” [43], which could lead to less overlap in CpGs between prediction models.

Unraveling the biological mechanisms underlying the gestational age clocks requires identifying the genes associated with the clock-specific CpGs and examining how they are related to gestational age. Our results revealed several genes in common across the different clocks. For example, 13 genes were shared between the EPIC GA clock and the Bohlin clock, while 15 genes were shared between the EPIC GA clock and the ETD-based clock. Some of the CpGs and genes in the EPIC GA clock appear to be stably associated with gestational age. For example, CpGs linked to Nuclear Receptor Corepressor 2 (*NCOR2)* and Insulin-Like Growth Factor 2 MRNA-binding protein 1 (*IGF2BP1)* were selected in both the EPIC GA clock and the Bohlin clock, and both of these genes have previously been identified in other studies of gestational age [44–47]. NCOR2 is involved in vitamin A metabolism and lung function [48], and IGF2BP1 plays an important role in embryogenesis and carcinogenesis [49]. The EPIC GA clock also identified CpGs related to Corticotropin-Releasing Factor-Binding Protein (*CRHBP)*, consistent with previous studies of gestational age [8, 50]. CRHBP levels rise throughout pregnancy but drop markedly when approaching term [51]. Furthermore, Mastorakos and Ilias [52] showed that CRHBP might prevent aberrant pituitary-adrenal stimulation in pregnancy. In addition to the genes mentioned here, several other genes linked to the CpGs in our clock have previously been implicated in gestational age, including Muscleblind Like Splicing Regulator 1 (*MBNL1*), CD82 molecule (*CD82*), Integrin Subunit Beta 2 (*ITGB2)*, and Rap Guanine Nucleotide Exchange Factor 3 (*RAPGEF3)* [47, 50]. Additional studies are needed to elucidate their roles in gestational age.

For a clock to be useful, it needs to be generalizable to other cohorts and populations. As with the Bohlin clock, our EPIC GA clock was trained on data from a relatively homogeneous cohort in terms of ethnicity, socioeconomic status, and age [32, 53]. Our clock performed equally well in the independent Finnish PREDO cohort. However, while the use of a homogeneous training set may enhance the prediction model [42, 54], it can also result in a cohort-specific clock that is less generalizable to other populations.

Exploring associations between specific neonatal outcomes and DNAm-based gestational age is still in its nascent stages [26, 55], and there are many unanswered questions regarding neonatal development. The development of an EPIC-specific gestational age clock may offer additional insights into gestational age and neonatal development. As the 450 K array has been discontinued, we anticipate that future research on DNAm-based GA clocks will migrate to the more updated EPIC array. Research on GA-related topics and DNAm utilizing the 450 K array are expected to continue for some time, as many 450 K-based datasets are still in circulation and some are being used in consortia-led efforts. The clocks presented here may facilitate further research on DNAm-based clocks for both 450 K and EPIC-based arrays.

## Conclusions

The new EPIC GA clock presented here predicted gestational age precisely in both ART and non-ART newborns and outperformed previous cord blood-based gestational age clocks when validated in an independent test set. The increased performance was not due to the higher coverage of CpGs on the EPIC array. Furthermore, the use of ETD-estimated gestational age for training did not improve the precision of gestational age prediction significantly compared with clocks trained on ultrasound-estimated gestational age. This is reassuring, as most datasets on newborns only have ultrasound- or LMP-based measures of gestational age. Finally, we did not find any significant association between GAA and ART. With a growing number of epigenetic datasets currently being generated on the EPIC platform, we expect our EPIC GA clock to become increasingly valuable in assessing developmental maturity in studies of neonatal development and disease.

## Methods

### Study population

MoBa is an ongoing, population-based pregnancy cohort study conducted by the Norwegian Institute of Public Health (NIPH). Totally, 114,500 children, 95,200 mothers, and 75,200 fathers were recruited from all over Norway from 1999 through 2008 [32]. The MoBa mothers consented to participation in 41% of the pregnancies. Extensive details on the MoBa cohort have been provided elsewhere [32, 56]. START is a substudy of MoBa and consists of 1,995 newborns and their parents. Blood samples from the newborns were obtained from the umbilical cord at birth [56].

PREDO is a prospective pregnancy cohort of Finnish women who gave birth to a singleton live child between 2006 and 2010 [33]. The cohort comprises 1079 pregnant women; 969 of these had one or more known risk factors for preeclampsia and intrauterine growth restriction, whereas the rest had no such risk factors. The women were enrolled in the study when they arrived for their first ultrasound screening at 12–14 gestational weeks in 10 study hospitals in Southern and Eastern Finland. Blood samples were obtained from the cord blood of 998 newborns [57]. To validate the gestational age clocks, we used cord blood-based DNAm data from 148 newborns (Fig. 1).

### DNAm profiling and quality control

Cord blood samples taken by a midwife immediately after birth were frozen [56]. Five hundred nanograms of DNA extracted from the cord blood of START newborns were shipped to LIFE & BRAIN GmbH in Bonn, Germany, for measurement of DNAm on the Illumina MethylationEPIC array (Illumina, San Diego, USA). The raw iDAT files were imported and processed in four batches using the R-package *RnBeads* [58]. 44,210 cross-hybridizing probes [59] and approximately 10,000 probes with a high detection p-value (above 0.01) were removed. 16,117 probes with the last three bases overlapping with a single-nucleotide polymorphism (SNP) were also excluded. The remaining DNAm signal was processed using BMIQ [60] to normalize the type I and type II probe chemistries. Control probes output from *RnBeads* were visually inspected for all samples, and those with low overall signals were removed. The *Greedycut* option [58] was used to remove outliers with markedly different DNAm signals than the rest of the samples. This resulted in the removal of 58 samples in total. For consistency, CpG sites removed from one batch, due to poor quality and detection p-value, were also removed from subsequent batches. After quality control, 770,586 autosomal CpGs and 1945 samples remained in the final dataset. 1793 subjects for whom we had information on ultrasound-based gestational age were used to develop and validate the gestational age clocks in this study.

For the PREDO samples, DNA was extracted according to standard procedures. Methylation analyses were performed at the Max Planck Institute of Psychiatry in Munich, Germany. DNA samples were bisulfite-converted using the EZ-96 DNA Methylation kit (Zymo Research, Irvine, CA) and assayed on the Illumina Infinium MethylationEPIC array (Illumina, San Diego, USA). Three samples were excluded for being outliers based on their median intensity values. Another three samples showing discordant phenotypic and estimated sex were excluded. A further three samples were contaminated with maternal DNA and were also removed [61]. Methylation beta-values were normalized using the *funnorm* function [62] in the R-package *minfi* [63]. Three samples showed density artifacts after normalization and were removed from further analysis. We excluded probes on the sex chromosomes, probes containing SNPs, and cross-hybridizing probes according to previously published criteria [59, 64, 65]. Furthermore, CpGs with a detection p-value > 0.01 in at least 25% of the samples were also excluded. Finally, one duplicate sample was removed after quality control. The final dataset contained 812,987 CpGs and 148 samples. After normalization, no significant batch effects were identified.

## Variables

For the START dataset, information on gestational age, sex, and ART status was extracted from the Medical Birth Registry of Norway (MBRN). Gestational age at birth was estimated by ultrasound measurements in week 18 of pregnancy. For the ART children, we used the date of egg retrieval plus 14 days to obtain a second estimate of gestational age. When the date of egg retrieval was not known, the date of embryo insertion was used instead, minus two days. For embryos that were frozen, we used the date of embryo insertion plus 14 days, and the number of days between egg retrieval and freezing. These three estimations of gestational age were combined into a variable called embryo transfer date (ETD). IVF and ICSI were defined as ART treatments, whereas children conceived by intrauterine insemination were defined as non-ART births.

For the PREDO dataset, information on gestational age and sex was extracted from the Finnish Medical Birth Register. Gestational age at birth was estimated by ultrasound measurements between 12 and 14 weeks of pregnancy.

### Gestational age prediction

Figure 1 shows a flowchart of the analyses performed. Children conceived without ART (non-ART) were randomly split into two groups: a training set (~ 80%) for developing the clock and a test set (~ 20%) for validating the clock. We used Lasso regression from the R-package *glmnet* [66] to develop DNAm-based predictors of gestational age. Clinically estimated gestational age was regressed on the 770,586 remaining CpGs after quality control in the START dataset. For the “450 K/EPIC overlap clock,” only the 397,473 CpGs that were in common between 450 K and EPIC were used. Missing probes were imputed using the median imputation procedure in the R-package *Hmisc* [67]. Tuning parameters α and λ were selected after tenfold cross-validation in the training set. For the “EPIC GA clock,” Lasso regression selected 176 CpGs (*α* = 1, *λ* = 0.66), while for the 450 K/EPIC overlap clock and the “ETD-based clock,” 173 CpGs (*α* = 1, *λ* = 0.63) and 156 CpGs (*α* = 1, *λ* = 0.62) were selected, respectively. Individual CpG sites and their corresponding coefficients are provided in Additional file 4.

The above clocks were used to estimate gestational age in (i) the START non-ART test set, (ii) the START ART newborns, and (iii) the non-ART newborns from PREDO (see Fig. 1 for more details). Predicted gestational age was regressed on clinically estimated gestational age using MM-type robust linear regression [68] from the R-package *robustbase* [69]. The precision of a given prediction model was defined as the proportion of variance explained by the model (i.e., by the R^2^ value). Accuracy, on the other hand, was defined as the median absolute deviation (MAD) between observed and predicted gestational age.

#### Comparison of prediction parameters

To compare the performances of the different clocks and GA estimation methods, we calculated the differences in R^2^, SE, and MAD when computed by two different clocks or GA methods. To assess the size and significance of the differences, we computed bootstrap confidence intervals for each difference. Since all three performance measures can be calculated from observed and predicted GA values, each bootstrap sample selected individuals randomly and used the observed and predicted GA values already calculated for those individuals. The pairs of R^2^, SE, and MAD values were calculated from the same bootstrap sample to account for the same dataset being used in each comparison. Thus, we did not need to refit the full prediction model for each bootstrap sample.

The bootstrapping was performed using the R-package *boot* [70, 71]. 95% confidence intervals of the bootstrap differences were standard percentile intervals, reported as type “perc” by the *boot* package. A difference was considered statistically significant when the corresponding confidence intervals did not include the value 0.

#### Gestational age acceleration analysis

GAA was defined as the residuals from a linear regression of DNAm gestational age predicted by the EPIC GA clock on ultrasound-estimated gestational age [16]. We tested for association between GAA and ART by performing a logistic regression of ART on GAA.

#### Gene-enrichment analysis

The online functional enrichment software WebGestalt [34] was used to search for enrichment within the annotated genes of the EPIC GA clock. We identified 154 unique gene names annotated for the 176 CpGs selected in the EPIC GA clock using the annotation data from Illumina’s Infinium MethylationEPIC v1.0 B4 Manifest file. We then performed an overrepresentation analysis on the 154 genes using Fisher’s exact test [35], assigning a minimum of five genes per category, and using the genome as background. WebGestalt leverages data from the following databases for each category: gene ontology [72, 73] (Biological Process, Cellular Component, Molecular Function), pathway (KEGG [74], Panther [75], Reactome [76], WikiPathway [77]), network (Kinase target, Transcription Factor target, miRNA target), disease (DisGeNET [78], GLAD4U [79], OMIM [80]), *drug* (DrugBank [81]), phenotype (Human Phenotype Ontology [82]), and chromosomal location (Cytogenic Band). The Benjamini–Hochberg procedure was applied to the p-values, and categories with a false discovery rate below 0.01 were declared significantly enriched.

## Supplementary Information

**Additional file 1: Figure S1**. This figure shows the prediction of gestational age in ART newborns using the ETD-clock.

**Additional file 2: Figure S2**. This figure shows the subgroup analysis of GAA in ART newborns with or without ICSI.

**Additional file 3: Figure S3**. This figure shows the subgroup analysis of GAA in ART newborns with fresh or frozen embryo transfer.

**Additional file 4**. This file includes the CpGs selected by the (A) EPIC GA, (B) 450K/EPIC overlap and (C) ETD-based clocks, their corresponding coefficients and annotated genes.

**Additional file 5**. This file includes the results from the WebGestalt analysis.

**Additional file 6**. This file includes the intercept and coefficients of the EPIC GA clock.

## Data Availability

MoBa data can be accessed by applying directly to NIPH; http://www.fhi.no/en/. Due to ethical issues and written consent, the PREDO datasets analyzed in the current study are not publicly available. However, interested researchers can obtain a de-identified dataset after approval from the PREDO Study Board. Data requests may be subject to further review by the national register authority and by the ethical committees. Data can be obtained upon reasonable request from the PREDO Study Board (predo.study@helsinki. fi) or individual researchers. The intercept, CpG sites, and coefficients for the EPIC GA clock can be found in Additional file 6. The clock can be applied to DNAm data using the following procedure: (1) generate a matrix of beta values (*n* individuals by *p* CpG sites), (2) select the CpG sites for the EPIC GA clock (Additional file 6) out of the matrix of beta values, (3) calculate the linear combination of the beta values and coefficients of the selected CpGs sites, and 4) add the intercept (Additional file 6) to the linear combination.

## Abbreviations

ART: Assisted reproductive technologies
DNAm: DNA methylation
27 K: Illumina HumanMethylation27
450 K: Illumina HumanMethylation450
EPIC: Illumina MethylationEPIC Bead Chip
LMP: Last menstrual period
ETD: Embryo transfer date
GAA: Gestational age acceleration
START: Study of Assisted Reproductive Technologies
MoBa: Norwegian Mother, Father and Child Cohort Study
PREDO: Prediction and Prevention of Preeclampsia and Intrauterine Growth Restriction
Lasso: Least absolute shrinkage and selection operator
US: Ultrasound
MAD: Median absolute deviation
CI: Confidence interval
IVF: In vitro fertilization
ICSI: Intracytoplasmic sperm injection
FDR: False discovery rate
NIPH: Norwegian Institute of Public Health
MBRN: Medical Birth Registry of Norway
REK: The Regional Committees for Medical and Health Research Ethics

## References

[1] Knight AK, Conneely KN, Smith AK. Gestational age predicted by DNA methylation: potential clinical and research utility. Epigenomics. 2017. 10.2217/epi-2016-0157 28111986

[2] Kerstjens JM, de Winter AF, Bocca-Tjeertes IF, Bos AF, Reijneveld SA (2012). Risk of developmental delay increases exponentially as gestational age of preterm infants decreases: a cohort study at age 4 years. Dev Med Child Neurol.

[3] Boyle EM, Poulsen G, Field DJ, Kurinczuk JJ, Wolke D, Alfirevic Z (2012). Effects of gestational age at birth on health outcomes at 3 and 5 years of age: population based cohort study. BMJ (Clinical research ed).

[4] Yuan W, Basso O, Sorensen HT, Olsen J (2001). Indicators of fetal growth and infectious disease in childhood–a birth cohort with hospitalization as outcome. Eur J Epidemiol.

[5] Kajantie E, Osmond C, Barker DJ, Eriksson JG (2010). Preterm birth–a risk factor for type 2 diabetes? The Helsinki birth cohort study. Diabetes Care.

[6] Bhutta AT, Cleves MA, Casey PH, Cradock MM, Anand KJ (2002). Cognitive and behavioral outcomes of school-aged children who were born preterm: a meta-analysis. JAMA.

[7] El Marroun H, Zeegers M, Steegers EA, van der Ende J, Schenk JJ, Hofman A (2012). Post-term birth and the risk of behavioural and emotional problems in early childhood. Int J Epidemiol.

[8] Simpkin AJ, Suderman M, Gaunt TR, Lyttleton O, McArdle WL, Ring SM (2015). Longitudinal analysis of DNA methylation associated with birth weight and gestational age. Hum Mol Genet.

[9] Hanson MA, Gluckman PD (2008). Developmental origins of health and disease: new insights. Basic Clin Pharmacol Toxicol.

[10] Mani S, Ghosh J, Coutifaris C, Sapienza C, Mainigi M (2020). Epigenetic changes and assisted reproductive technologies. Epigenetics.

[11] Morgan HD, Santos F, Green K, Dean W, Reik W. Epigenetic reprogramming in mammals. Hum Mol Genet. 2005;14 Spec No 1:R47–58. 10.1093/hmg/ddi114 15809273

[12] von Meyenn F, Reik W (2015). Forget the parents: epigenetic reprogramming in human germ cells. Cell.

[13] Messerschmidt DM, Knowles BB, Solter D (2014). DNA methylation dynamics during epigenetic reprogramming in the germline and preimplantation embryos. Genes Dev.

[14] Zhou F, Wang R, Yuan P, Ren Y, Mao Y, Li R (2019). Reconstituting the transcriptome and DNA methylome landscapes of human implantation. Nature.

[15] Bohlin J, Haberg SE, Magnus P, Reese SE, Gjessing HK, Magnus MC (2016). Prediction of gestational age based on genome-wide differentially methylated regions. Genome Biol.

[16] Knight AK, Craig JM, Theda C, Baekvad-Hansen M, Bybjerg-Grauholm J, Hansen CS (2016). An epigenetic clock for gestational age at birth based on blood methylation data. Genome Biol.

[17] Mayne BT, Leemaqz SY, Smith AK, Breen J, Roberts CT, Bianco-Miotto T (2017). Accelerated placental aging in early onset preeclampsia pregnancies identified by DNA methylation. Epigenomics.

[18] Lee Y, Choufani S, Weksberg R, Wilson SL, Yuan V, Burt A (2019). Placental epigenetic clocks: estimating gestational age using placental DNA methylation levels. Aging.

[19] Pidsley R, Zotenko E, Peters TJ, Lawrence MG, Risbridger GP, Molloy P (2016). Critical evaluation of the Illumina MethylationEPIC BeadChip microarray for whole-genome DNA methylation profiling. Genome Biol.

[20] Dhingra R, Kwee LC, Diaz-Sanchez D, Devlin RB, Cascio W, Hauser ER (2019). Evaluating DNA methylation age on the Illumina MethylationEPIC Bead Chip. PLoS ONE.

[21] Skalkidou A, Kullinger M, Georgakis MK, Kieler H, Kesmodel US (2018). Systematic misclassification of gestational age by ultrasound biometry: implications for clinical practice and research methodology in the Nordic countries. Acta Obstet Gynecol Scand.

[22] Gjessing HK, Grottum P, Eik-Nes SH (2007). A direct method for ultrasound prediction of day of delivery: a new, population-based approach. Ultrasound Obstet Gynecol.

[23] Delpachitra P, Palmer K, Onwude J, Meagher S, Rombauts L, Waalwyk K (2012). Ultrasound reference chart based on IVF dates to estimate gestational age at 6–9 weeks' gestation. ISRN Obstet Gynecol.

[24] Girchenko P, Lahti J, Czamara D, Knight AK, Jones MJ, Suarez A (2017). Associations between maternal risk factors of adverse pregnancy and birth outcomes and the offspring epigenetic clock of gestational age at birth. Clin Epigenet.

[25] Palma-Gudiel H, Eixarch E, Crispi F, Morán S, Zannas AS, Fañanás L (2019). Prenatal adverse environment is associated with epigenetic age deceleration at birth and hypomethylation at the hypoxia-responsive EP300 gene. Clin Epigenet.

[26] Khouja JN, Simpkin AJ, O'Keeffe LM, Wade KH, Houtepen LC, Relton CL (2018). Epigenetic gestational age acceleration: a prospective cohort study investigating associations with familial, sociodemographic and birth characteristics. Clin Epigenet.

[27] Cavoretto P, Candiani M, Giorgione V, Inversetti A, Abu-Saba MM, Tiberio F (2018). Risk of spontaneous preterm birth in singleton pregnancies conceived after IVF/ICSI treatment: meta-analysis of cohort studies. Ultrasound Obstet Gynecol.

[28] Helmerhorst FM, Perquin DA, Donker D, Keirse MJ (2004). Perinatal outcome of singletons and twins after assisted conception: a systematic review of controlled studies. BMJ (Clinical research ed).

[29] Kalra SK, Barnhart KT. In vitro fertilization and adverse childhood outcomes: what we know, where we are going, and how we will get there: a glimpse into what lies behind and beckons ahead. Fertil Steril. 2011;95(6):1887–9. 10.1016/j.fertnstert.2011.02.044 PMC3080444 21411083

[30] Pandey S, Shetty A, Hamilton M, Bhattacharya S, Maheshwari A (2012). Obstetric and perinatal outcomes in singleton pregnancies resulting from IVF/ICSI: a systematic review and meta-analysis. Hum Reprod Update.

[31] El Hajj N, Haertle L, Dittrich M, Denk S, Lehnen H, Hahn T (2017). DNA methylation signatures in cord blood of ICSI children. Human Reprod (Oxford, England).

[32] Magnus P, Birke C, Vejrup K, Haugan A, Alsaker E, Daltveit AK (2016). Cohort profile update: the Norwegian mother and child cohort study (MoBa). Int J Epidemiol.

[33] Girchenko P, Lahti M, Tuovinen S, Savolainen K, Lahti J, Binder EB (2017). Cohort profile: prediction and prevention of preeclampsia and intrauterine growth restriction (PREDO) study. Int J Epidemiol.

[34] Liao Y, Wang J, Jaehnig EJ, Shi Z, Zhang B (2019). WebGestalt 2019: gene set analysis toolkit with revamped UIs and APIs. Nucleic Acids Res.

[35] Fisher RA (1922). On the interpretation of *χ*_2_ from contingency tables, and the calculation of P. J R Stat Soc.

[36] Heintzman ND, Ren B (2009). Finding distal regulatory elements in the human genome. Curr Opin Genet Dev.

[37] Lee Y, Haftorn KL, Denault WRP, Nustad HE, Page CM, Lyle R (2020). Blood-based epigenetic estimators of chronological age in human adults using DNA methylation data from the Illumina MethylationEPIC array. BMC Genom.

[38] Melamed N, Choufani S, Wilkins-Haug LE, Koren G, Weksberg R (2015). Comparison of genome-wide and gene-specific DNA methylation between ART and naturally conceived pregnancies. Epigenetics.

[39] Novakovic B, Lewis S, Halliday J, Kennedy J, Burgner DP, Czajko A (2019). Assisted reproductive technologies are associated with limited epigenetic variation at birth that largely resolves by adulthood. Nat Commun.

[40] White CR, Denomme MM, Tekpetey FR, Feyles V, Power SG, Mann MR (2015). High frequency of imprinted methylation errors in human preimplantation embryos. Sci Rep.

[41] Bergsma T, Rogaeva E (2020). DNA methylation clocks and their predictive capacity for aging phenotypes and healthspan. Neurosci Insights.

[42] Engebretsen S, Bohlin J (2019). Statistical predictions with glmnet.. Clin Epigenet.

[43] Zou H, Hastie T. Regularization and variable selection via the elastic net. 2005;67(2):301-20.

[44] Merid SK, Novoloaca A, Sharp GC, Küpers LK, Kho AT, Roy R (2020). Epigenome-wide meta-analysis of blood DNA methylation in newborns and children identifies numerous loci related to gestational age. Genome Med.

[45] Cruickshank MN, Oshlack A, Theda C, Davis PG, Martino D, Sheehan P (2013). Analysis of epigenetic changes in survivors of preterm birth reveals the effect of gestational age and evidence for a long term legacy. Genome Med.

[46] Fernando F, Keijser R, Henneman P, van der Kevie-Kersemaekers AM, Mannens MM, van der Post JA (2015). The idiopathic preterm delivery methylation profile in umbilical cord blood DNA. BMC Genom.

[47] Wang XM, Tian FY, Fan LJ, Xie CB, Niu ZZ, Chen WQ (2019). Comparison of DNA methylation profiles associated with spontaneous preterm birth in placenta and cord blood. BMC Med Genom.

[48] Minelli C, Dean CH, Hind M, Alves AC, Amaral AF, Siroux V (2016). Association of forced vital capacity with the developmental gene NCOR2. PLoS ONE.

[49] Huang X, Zhang H, Guo X, Zhu Z, Cai H, Kong X (2018). Insulin-like growth factor 2 mRNA-binding protein 1 (IGF2BP1) in cancer. J Hematol Oncol.

[50] Schroeder JW, Conneely KN, Cubells JC, Kilaru V, Newport DJ, Knight BT (2011). Neonatal DNA methylation patterns associate with gestational age. Epigenetics.

[51] Perkins AV, Eben F, Wolfe CD, Schulte HM, Linton EA (1993). Plasma measurements of corticotrophin-releasing hormone-binding protein in normal and abnormal human pregnancy. J Endocrinol.

[52] Mastorakos G, Ilias I (2003). Maternal and fetal hypothalamic-pituitary-adrenal axes during pregnancy and postpartum. Ann N Y Acad Sci.

[53] Nilsen RM, Vollset SE, Gjessing HK, Skjaerven R, Melve KK, Schreuder P (2009). Self-selection and bias in a large prospective pregnancy cohort in Norway. Paediatr Perinat Epidemiol.

[54] Simpkin AJ, Suderman M, Howe LD (2017). Epigenetic clocks for gestational age: statistical and study design considerations. Clin Epigenetics.

[55] Knight AK, Smith AK, Conneely KN, Dalach P, Loke YJ, Cheong JL (2018). Relationship between epigenetic maturity and respiratory morbidity in preterm infants. J Pediatr.

[56] Paltiel L, Anita H, Skjerden T, Harbak K, Bækken S, Nina Kristin S, et al. The biobank of the Norwegian Mother and Child Cohort Study—present status. Norsk Epidemiologi. 2014;24(1–2).

[57] Czamara D, Eraslan G, Page CM, Lahti J, Lahti-Pulkkinen M, Hämäläinen E (2019). Integrated analysis of environmental and genetic influences on cord blood DNA methylation in new-borns. Nat Commun.

[58] Muller F, Scherer M, Assenov Y, Lutsik P, Walter J, Lengauer T, et al. RnBeads 2.0: comprehensive analysis of DNA methylation data. Genome Biol. 2019;20(1):55. 10.1186/s13059-019-1664-9 PMC6419383 30871603

[59] McCartney DL, Walker RM, Morris SW, McIntosh AM, Porteous DJ, Evans KL (2016). Identification of polymorphic and off-target probe binding sites on the Illumina Infinium MethylationEPIC BeadChip. Genomics data.

[60] Teschendorff AE, Marabita F, Lechner M, Bartlett T, Tegner J, Gomez-Cabrero D (2013). A beta-mixture quantile normalization method for correcting probe design bias in Illumina Infinium 450 k DNA methylation data. Bioinformatics (Oxford, England).

[61] Morin AM, Gatev E, McEwen LM, MacIsaac JL, Lin DTS, Koen N (2017). Maternal blood contamination of collected cord blood can be identified using DNA methylation at three CpGs. Clin Epigenet.

[62] Fortin JP, Labbe A, Lemire M, Zanke BW, Hudson TJ, Fertig EJ (2014). Functional normalization of 450k methylation array data improves replication in large cancer studies. Genome Biol.

[63] Aryee MJ, Jaffe AE, Corrada-Bravo H, Ladd-Acosta C, Feinberg AP, Hansen KD (2014). Minfi: a flexible and comprehensive Bioconductor package for the analysis of Infinium DNA methylation microarrays. Bioinformatics (Oxford, England).

[64] Chen YA, Lemire M, Choufani S, Butcher DT, Grafodatskaya D, Zanke BW (2013). Discovery of cross-reactive probes and polymorphic CpGs in the Illumina Infinium HumanMethylation450 microarray. Epigenetics.

[65] Price ME, Cotton AM, Lam LL, Farré P, Emberly E, Brown CJ (2013). Additional annotation enhances potential for biologically-relevant analysis of the Illumina Infinium HumanMethylation450 BeadChip array. Epigenet Chromatin.

[66] Friedman J, Hastie T, Tibshirani R (2010). Regularization paths for generalized linear models via coordinate descent. J Stat Softw.

[67] Harrell Jr. FE. Hmisc: Harrell Miscellaneous. R package 4.4–1 ed. https://CRAN.R-project.org/package=Hmisc 2020.

[68] Yohai V. High breakdown-point and high efficiency robust estimates for regression. Ann Stat. 1987;15.

[69] Maechler M RP, Croux C, Todorov V, Ruckstuhl A, Salibian-Barrera M, Verbeke T, Koller M, Conceicao EL, Anna di Palma M. robustbase: Basic robust statistics. R package 0.93–6 ed. http://robustbase.r-forge.r-project.org/. 2020.

[70] Canty A. RBD. boot: Boostrap R (S-Plus) Functions. R package version 1.3–25 ed. https://CRAN.R-project.org/package=boot2020.

[71] Davison AC, Hinkley DV (1997). Bootstrap methods and their application.

[72] Ashburner M, Ball CA, Blake JA, Botstein D, Butler H, Cherry JM (2000). Gene ontology: tool for the unification of biology. Gene Ontol Consortium Nat Genet.

[73] The Gene Ontology Resource: 20 years and still GOing strong. Nucleic Acids Res. 2019;47(D1):D330-d8. 10.1093/nar/gky1055 PMC6323945 30395331

[74] Kanehisa M, Sato Y, Kawashima M, Furumichi M, Tanabe M (2015). KEGG as a reference resource for gene and protein annotation. Nucleic Acids Res.

[75] Thomas PD, Campbell MJ, Kejariwal A, Mi H, Karlak B, Daverman R (2003). PANTHER: a library of protein families and subfamilies indexed by function. Genome Res.

[76] Jassal B, Matthews L, Viteri G, Gong C, Lorente P, Fabregat A (2020). The reactome pathway knowledgebase. Nucleic Acids Res.

[77] Kutmon M, Riutta A, Nunes N, Hanspers K, Willighagen EL, Bohler A (2016). WikiPathways: capturing the full diversity of pathway knowledge. Nucleic Acids Res.

[78] Piñero J, Ramírez-Anguita JM, Saüch-Pitarch J, Ronzano F, Centeno E, Sanz F (2020). The DisGeNET knowledge platform for disease genomics: 2019 update. Nucleic Acids Res.

[79] Jourquin J, Duncan D, Shi Z, Zhang B. GLAD4U: deriving and prioritizing gene lists from PubMed literature. BMC Genom. 2012;13 Suppl 8(Suppl 8):S20. 10.1186/1471-2164-13-S8-S20 PMC3535723 23282288

[80] Online Mendelian Inheritance in Man, OMIM®: McKusick-Nathans Institute of Genetic Medicine, Johns Hopkins University (Baltimore, MD); [20.05.2020]. Available from: httos://omim.org/.

[81] Wishart DS, Knox C, Guo AC, Shrivastava S, Hassanali M, Stothard P, et al. DrugBank: a comprehensive resource for in silico drug discovery and exploration. Nucleic Acids Res. 2006;34(Database issue):D668–72. 10.1093/nar/gkj067 PMC1347430 16381955

[82] Köhler S, Carmody L, Vasilevsky N, Jacobsen JOB, Danis D, Gourdine JP (2019). Expansion of the human phenotype ontology (HPO) knowledge base and resources. Nucleic Acids Res.

