# Supplementary figures and images for: An EPIC predictor of gestational age and its application to newborns conceived by assisted reproductive technologies
Source: Clin Epigenetics. 2021 Apr 19;13:82. doi: 10.1186/s13148-021-01055-z (PMC8056641; doi:10.1186/s13148-021-01055-z)

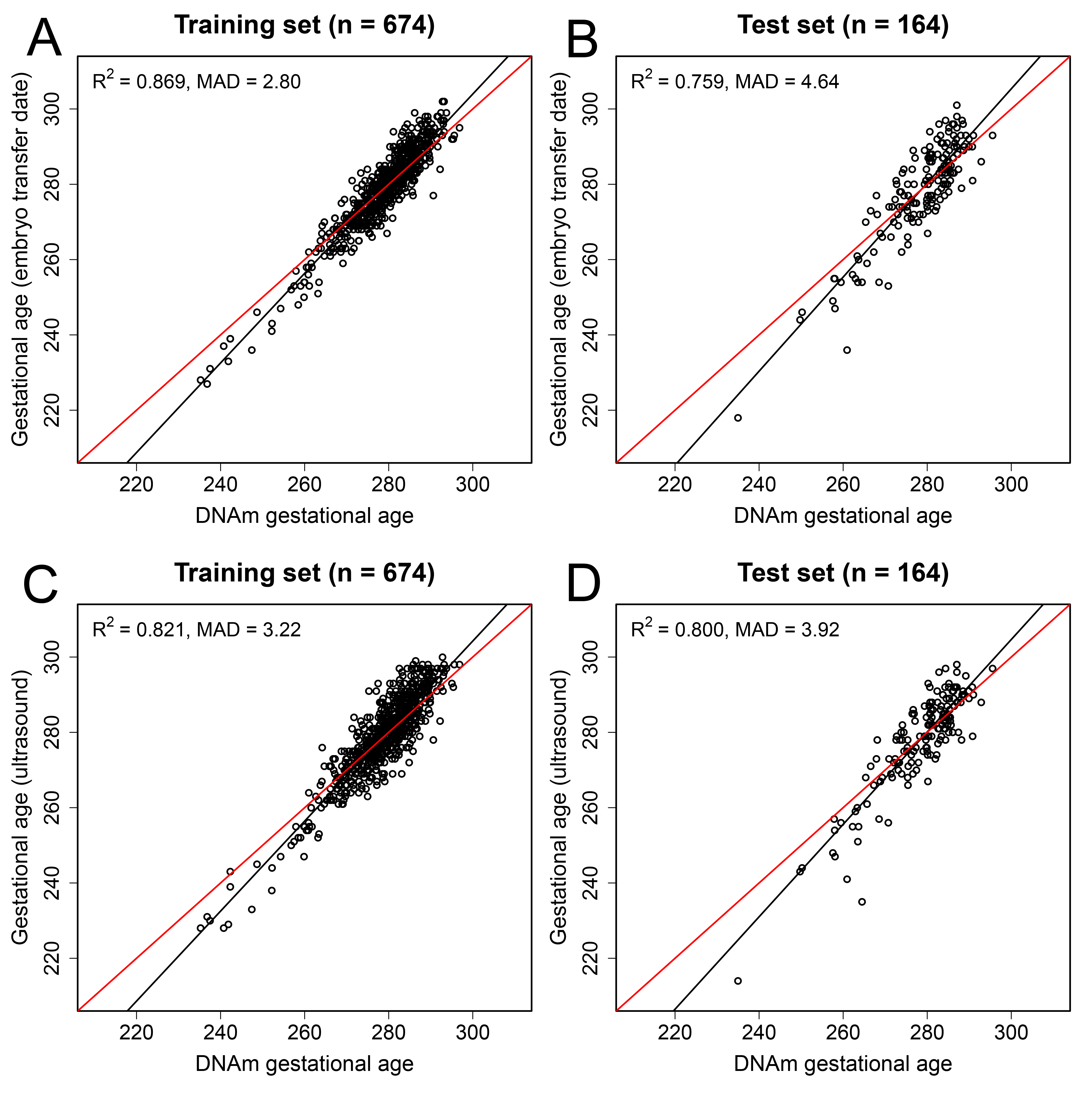

Supplement: Supplementary file 1 — Additional file 1: Figure S1. This figure shows the prediction of gestational age in ART newborns using the ETD-clock. [file 13148_2021_1055_MOESM1_ESM.tif]

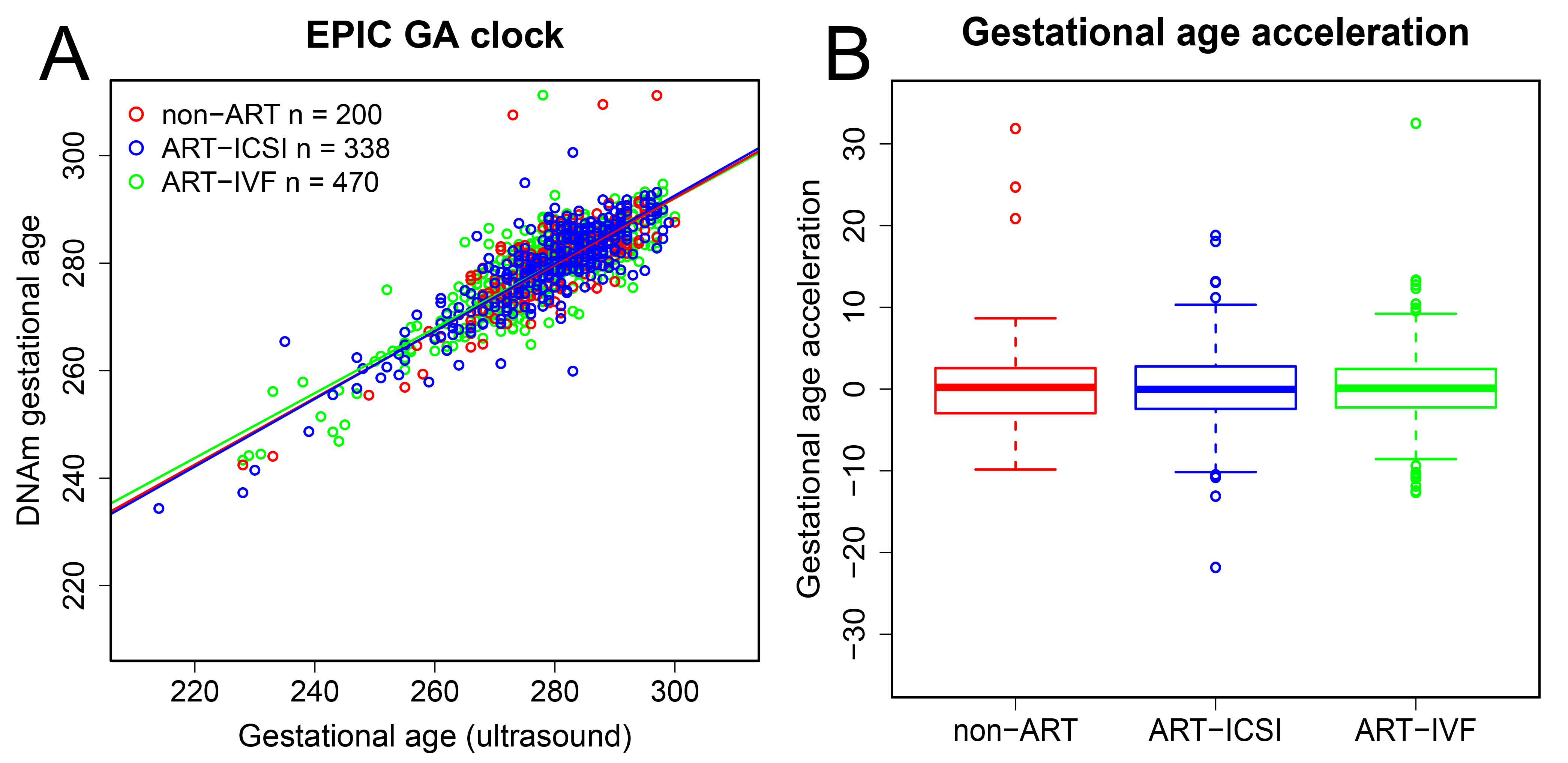

Supplement: Supplementary file 2 — Additional file 2: Figure S2. This figure shows the subgroup analysis of GAA in ART newborns with or without ICSI. [file 13148_2021_1055_MOESM2_ESM.tif]

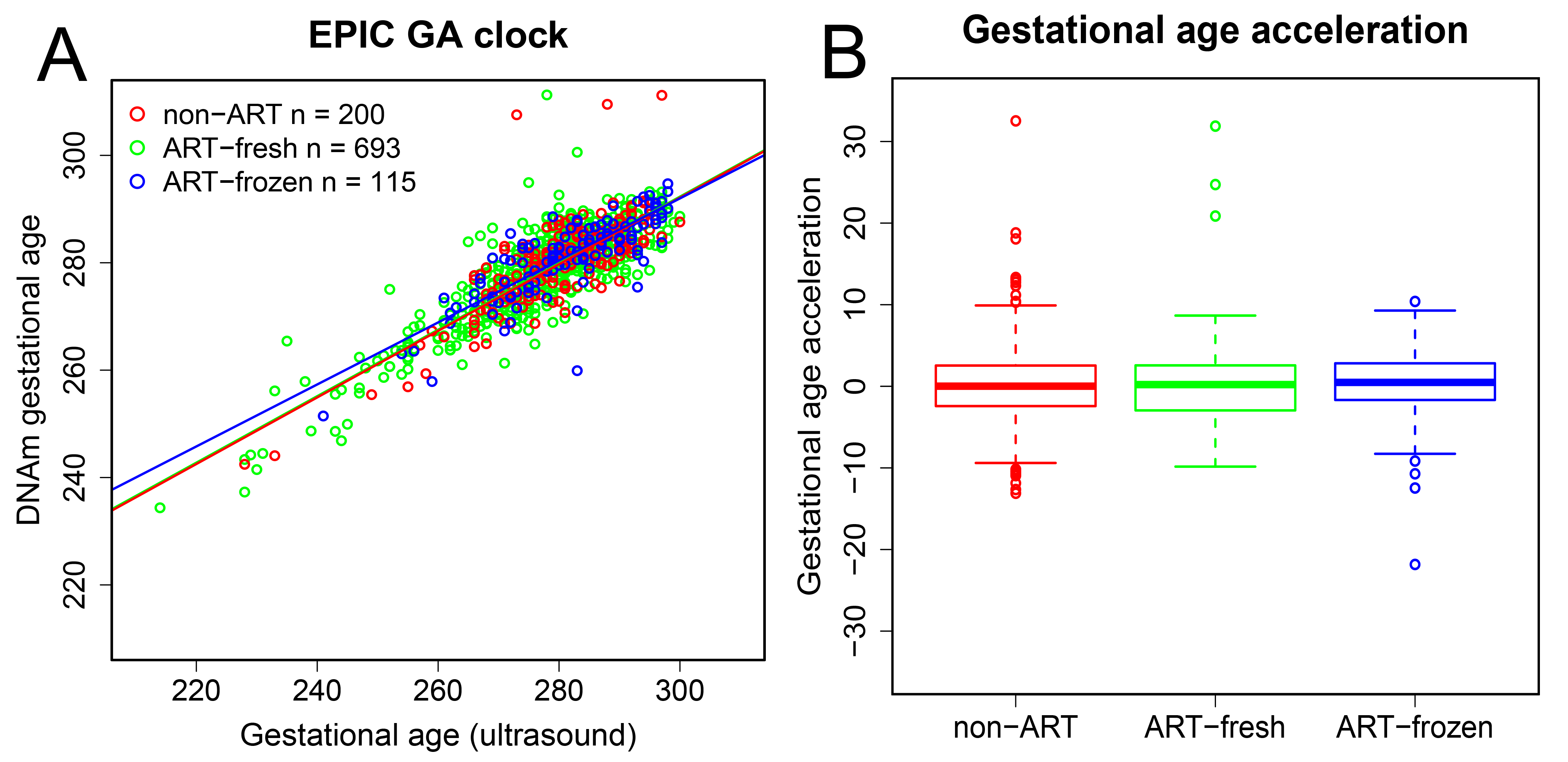

Supplement: Supplementary file 3 — Additional file 3: Figure S3. This figure shows the subgroup analysis of GAA in ART newborns with fresh or frozen embryo transfer. [file 13148_2021_1055_MOESM3_ESM.tif]
